# Footwear effect on the orthopedic injury profile in ankle and foot trauma caused by motorcycle accidents

**DOI:** 10.1590/1413-785220263403e292429

**Published:** 2026-05-29

**Authors:** Hugo Leonardo Neri de Lima, Maria Josycley Novais Landim Soares, Sally Mariah de Morais Pinheiro Cruz Macedo, Marcelo Parente Oliveira

**Affiliations:** 1 Universidade Federal do Cariri, Barbalha, CE, Brazil.

**Keywords:** Lower limbs, Shoes, Wounds and injuries, Traffic accidents, Motorcycles, Membros inferiores, Calçados, Ferimentos e lesões, Acidentes de trânsito, Motocicletas

## Abstract

Motorcycle accidents represent a significant cause of trauma in developing countries such as Brazil. Within the possible lesions, lower limb injuries are among the most prevalent, with ankle and foot injuries causing a high incidence of morbidity and long-term sequelae. This may partly be due to the low use of protective footwear. Therefore, this study investigated the relationship between the prevalence and severity of lower limb injuries at the ankle and foot level in motorcycle accident victims and the use or non-use of appropriate footwear. To this end, data collection was conducted in the emergency department and inpatient unit of a trauma reference hospital in the interior of the state of Ceará, using a form, clinical images, clinical examinations of injuries, and information from personal medical records over a two-month period. The results showed a significant association between inadequate use of protective footwear and an increase in the frequency and severity of soft tissue injuries. Additionally, the use of closed protective footwear covering the ankle demonstrated a noteworthy protective value. However, no significant relationship was found between footwear use and the presence or severity of fractures, hospitalization, or the need for surgical procedures. **Level of Evidence II; Retrospective Study**.

## RESUMO

Acidentes motociclísticos correspondem a importante causa de trauma em países em desenvolvimento como o Brasil. Dentre traumas possíveis, lesões em membros inferiores estão entre as mais prevalentes, nas quais lesões em tornozelos e pés provocam alta incidência de morbidade e sequelas no decorrer da vida. Parte disso pode estar relacionado com a baixa taxa de uso de calçados protetivos. Assim, este estudo verificou a relação entre a prevalência e gravidade de lesões em membros inferiores ao nível de tornozelos e pés, em pacientes vítimas de acidente de motocicleta e o uso ou não de calçados adequados. Para isto, foi feito levantamento na urgência e enfermaria de um hospital referência em atendimento ao trauma no interior do estado do Ceará, por meio de formulário, imagens e exame clínico de lesões, e de informações de prontuário pessoal durante o período de dois meses. Os resultados mostraram relação importante entre o uso de calçados sem proteção adequada com aumento e gravidade de lesões de partes moles, e valor protetivo relevante com uso de sapatos com proteção fechada e cobertura de tornozelo, mas sem relação significativa com presença e gravidade de fraturas, internamento ou necessidade de procedimentos cirúrgicos. **Nível de Evidência II; Estudo Retrospectivo**.

## INTRODUCTION

Motorcycle accidents are one of the leading causes of trauma that arrive at emergency rooms worldwide. In Iran, motorcycles were involved in 43.4% of accidents.^1^ In Africa, motorcycle accidents account for 57% of all traffic accidents.^2^

In Brazil, there is a trend of a linear increase in motorcyclist mortality across all regions, with the greatest annual variation in the Northeast region.^3^ Data from the SIH (Hospital Information System) in 2013 showed that among all hospitalizations resulting from traffic accidents, 51.9% were caused by motorcycle accidents.^4^

Data shows that motorcyclists have a higher chance of losing their lives in accidents, and are eight to fourteen times more likely to suffer any type of injury compared to victims of accidents involving cars and other means of transportation.^5,6^

Injuries to the lower limbs are the most common among motorcycle accidents, affecting between 30-70% of victims.^7-9^

Although the most common injuries are to the lower limbs^2^, individuals, in general, give little importance to the use of other types of protection besides helmets, as verified in a multicenter African study by Ngunde in 2019,^10^ which found a 47.93% prevalence of lower limb injury (10.66% bilateral), 12.69% in the ankle and feet, 60.29% of the total classified as exposed injuries, and among the victims, little or no protection on extremities was verified.

Previous research has already verified that the use of personal protective equipment, in addition to wearing a helmet, such as appropriate footwear, provides protection against injuries.^11^

Taking this into consideration, the objective was to seek a relationship between the presence and severity of injuries in the lower limbs at the ankle and foot level in individuals who are victims of motorcycle accidents, with the use or non-use of various types of footwear.

## METHODOLOGY

Clinical and radiographic data were collected, both in the emergency room and wards, from 72 patients who were victims of motorcycle accidents with injuries to their ankles and/or feet over a period of 2 months, from October to November 2024, treated at a large hospital unit in the state of Ceará (Brazil), a reference in orthopedic trauma. The study was approved by the ethics committee of the Federal University of Cariri (UFCA) on October 1, 2024, under the CAAE (Certificate of Ethical Appreciation Presentation) No. 79386424.4.0000.5698. All participants signed a Free and Informed Consent Term, duly reviewed according to CAAE. Patients who refused to provide information about their footwear at the time of the accident or who refused to sign the consent terms for participation and for information in the medical record were excluded.

The data were organized in a Microsoft Office Excel® spreadsheet, developed by Microsoft Corporation (WA-USA), and analyzed using SPSS software version 24. For qualitative variables, absolute and relative frequencies were calculated. Quantitative variables were summarized using statistics: mean, standard deviation, and quartiles. Qualitative variables regarding the type of footwear were tested using Fisher's exact test, while quantitative variables were tested using Mann-Whitney and Kruskal-Wallis tests. For all inferential procedures, a significance level of 5% was adopted.

**Figure 1:**
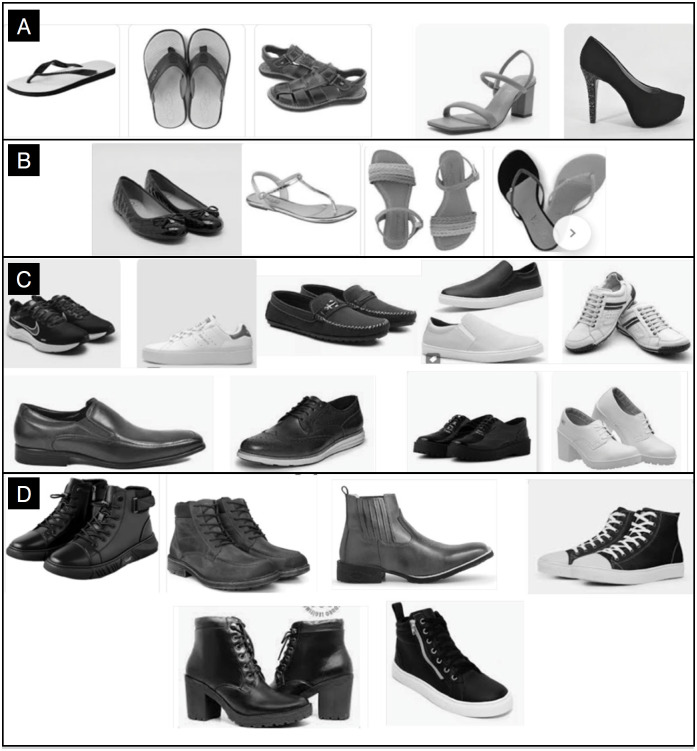
FORM. A. Barefoot. B. Footwear without covering: Flip-flops, sandals, espadrilles, flat sandals, open-toe high heels, ballet flats without covering. C. Footwear with low cut/covering (up to ankle height): Sneakers, dress footwears, casual footwears, sports footwears, low-cut boots. D. Footwear with high cut/covering (above the ankle): Work boots, motorcycle boots, high-top sneakers.

It was verified for each patient which footwear was used during the accident through an objective form with images/models of the types of footwear, determining 4 groups: type A – absence of any type of footwear; type B – presence of footwear such as flip-flops or sandals, without protection for the dorsum of the foot and ankle, and without any means of securing to the foot; type C – closed footwear with means of securing to the foot and with protection up to the ankle level; and type D – closed footwear like group C, providing protection above the ankle (Figure 1). Injuries were classified during the first care, and if already hospitalized, in the ward bed, as follows: 1 – Soft tissue injuries according to the Tscherne^12^ classification and by AOSTC (AO Soft-Tissue Classification) – IC (Closed Injury) and OI (Open Injury); 2 – Closed fractures by the AO/OTA Fracture and Dislocation Classification (2018);^13^ 3 – Open fractures by the Gustillo and Anderson Classification.^14^ Radiographic data were collected from the hospital database. Other necessary information was collected from the physical or electronic medical record.

## RESULTS

Data were collected from 72 patients with orthopedic injuries related to motorcycle accidents who met the study criteria.

There was a dominance of male patients (77.8%). The average age in the sample was 34.2 years (+/- 14.5). The summaries of the Epidemiological data are available in Table 1.

**Table 1:** Distribution of frequencies of sex and age group among participants.

Variables	n	%
**Gender**		
Male	56	77.8
Female	16	22.2
**Age range**		
15 to 19	11	15.3
20 to 29	22	30.6
30 to 39	16	22.2
40 to 49	10	13.9
50 to 59	9	12.5
60 or more	4	5.6
Mean ± standard deviation	34.2 ± 14.5	
Median (1st - 3rd quartile)	31.0 (22.5 - 45.0)	
Minimum - maximum	15 – 71	

Regarding footwear, type B was most prevalent (62.5%), followed by type C (22.2%), D (12.5%), and, lastly, 2 participants were without footwear – Type A (2.8%) (Table 2).

**Table 2:** Percentage evaluation of footwear type, injury type, injury classifications, hospitalizations, and surgical procedures.

Variables	n	%
**Footwear type**		
A	2	2.8
B	45	62.5
C	16	22.2
D	9	12.5
**Fracture and/or dislocation with/without open fracture**		
Yes	28	38.9
No	44	61.1
**Exposed injury with/without fracture**		
Yes	23	31.9
No	49	68.1
**Closed soft tissue injury with/without fracture**		
Yes	49	68.0
No	23	32.0
**Fractures and/or dislocations according to location in the participant (n=28)**		
Distal tibia	6	21.4
Ankle	5	17.9
Hindfoot	0	0
Midfoot	0	0
Forefoot	17	60.7
**Classification of closed soft tissue injuries according to Tscherne (n=49)**		
0	10	20.4
1	31	63.3
2	8	16.3
3	0	0
**Classification of Fractures according to AO-OTA (n=35)**		
Type A	19	54.3
Type B	8	22.9
Type C or greater	8	22.9
**Classification of exposed fractures according to Gustillo and Anderson (n=15)**		
1	3	20.0
2	7	46.7
3rd	5	33.3
3B	0	0
3C	0	0
**Classification of closed soft tissue injuries according to AOSTC - IC (n=49)**		
1	11	22.4
2	27	55.1
3	10	20.4
4	1	2.0
5	0	0
**Classification of exposed injuries according to AOSTC - IO (n=23)**		
1	2	8.7
2	10	43.5
3	5	21.7
4	5	21.7
5	1	4.3
Sprain		
Yes	25	34.7
No	47	65.3
**Grade of sprain (n=25)**		
Grade 1	9	36.0
Grade 2	16	64.0
Grade 3	0	0
**Internment**		
Yes	28	38.9
No	44	61.1
**Surgical Procedure**		
Yes	32	44.4
No	40	55.6

There were 49 (68%) participants with closed soft tissue injuries with or without fractures, of which the majority were in group A/B (41.7%), 11 (15.3%) were in group C, and 8 (11.1%) were from group D. There was no significant relationship between the presence of soft tissue injuries and the type of footwear used (p = 0.335). Among the type A footwear, all injuries were exposed (Table 3).

**Table 3:** Percentage evaluation of the distribution of fractures/dislocations/soft tissue injuries and surgical procedures by type of footwear.

Groups	N	%
**Number of fractures/dislocations by type of footwear**		
A/B	16	45.7
C	15	42.8
D	4	5.5
Total	35	
**Number of exposed fractures/dislocations by type of footwear**		
A/B	9	50
C	8	44.4
D	1	5.6
Total	18	
**Number of surgical procedures according to the type of footwear**		
A/B	26	61.9
C	13	30.9
D	3	7.1
Total	42	
**Number of closed soft tissue injuries by type of footwear**		
A/B	30	41.7
C	11	15.3
D	8	11.1
Total	49	

Sprains were recorded in 25 participants (34.7%) (Table 3), with patients wearing type A/B footwear showing the highest proportion (40.4%) and the lowest in type D (22.2%), but without a statistically significant difference (p = 0.458) (Table 4).

**Table 4:** Statistical evaluation regarding the type and classification of injuries, occurrence of hospitalization and surgical procedure according to the type of footwear of the participants.

Variables	Footwear A/B		Footwear C		Footwear D		p-value
	n	%	n	%	n	%	
**Fracture and/or dislocation with or without open fracture**							**0.484**
Yes	16	34.0	8	50.0	4	44.4	
No	31	66.0	8	50.0	5	55.6	
**Exposed injury with/without fracture**							**0.388**
Yes	17	36.2	5	31.3	1	11.1	
No	30	63.8	11	68.8	8	88.9	
**Closed soft tissue injury with/without fracture**							**0.335**
Yes	30	63.8	11	68.7	8	88.9	
No	17	36.2	5	31.3	1	11.1	
**AOSTC Classification - IC (n=49)**							**0.004**
1	2	6.7	6	54.5	3	37.5	
2	17	56.7	5	45.5	5	62.5	
3	10	33.3	0	0.0	0	0.0	
4	1	3.3	0	0.0	0	0.0	
**AOSTC Classification - IO (n=23)**							**0.308**
1	1	5.9	1	20.0	0	0.0	
2	8	47.1	2	40.0	0	0.0	
3	2	11.8	2	40.0	1	100.0	
4	5	29.4	0	0.0	0	0.0	
5	1	5.9	0	0.0	0	0.0	
**Tscherne Classification (n=49)**							**<0.001**
0	1	3.3	6	54.5	3	37.5	
1	21	70.0	5	45.5	5	62.5	
2	8	26.7	0	0.0	0	0.0	
**Gustillo and Anderson Classification (n=15)**							**0.895**
1	2	22.2	1	20.0	0	0.0	
2	4	44.4	3	60.0	0	0.0	
3A	3	33.3	1	20.0	1	100.0	
**Sprain**							**0.458**
Yes	19	40.4	4	25.0	2	22.2	
No	28	59.6	12	75.0	7	77.8	
**Grade of sprain (n=25)**							**0.803**
Grade 1	6	31.6	2	50.0	1	50.0	
Grade 2	13	68.4	2	50.0	1	50.0	
**Hospitalization**							**0.393**
Yes	18	38.3	8	50.0	2	22.2	
No	29	61.7	8	50.0	7	77.8	
**Surgical procedure**							**0.432**
Yes	22	46.8	8	50.0	2	22.2	
No	25	53.2	8	50.0	7	77.8	

Fisher's exact test.

When classified according to Tscherne, type A/B footwears showed a significant relationship with grade 1 injuries (63.3%) and grade 2 injuries (26.7%) (p < 0.001) (Table 4), whereas type C and D footwears showed no grade 2 or higher injuries. And when classified according to AO/STC - IC, with the majority of injuries being grade 2 (55.1%), there was a significant relationship (p = 0.004) for a higher number and higher grade of injuries in patients with A/B footwears, while patients with type C and D footwears showed a higher correlation with lower grade injuries (STC - IC 1).

Regarding the presence of fractures and/or dislocations with or without open fracture, a total of 35 fractures and/or dislocations were verified, distributed among 28 (38.9%) of the participants (Table 3). These injuries did not show a significant relationship according to the type of footwear (p = 0.484), as shown in Table 4. Fractures of the forefoot predominated (23.6%), with no fractures of the midfoot and hindfoot observed (Table 2).

The fractures were mostly classified according to AO-OTA as type A (54.3%) (Table 2), and the most affected area among participants was the forefoot (60.7%).

23 patients with exposed injuries (with or without fractures) were classified (31.9%), with a relative predominance of incidence present in the A/B footwear groups, 36.2% (p = 0.388) (Table 4). When classified by AO/STC – IO, there was a predominance of grade 2 injuries (43.5%), with most related to patients with type A/B footwears (47.1%) (p = 0.308).

Regarding exposed fractures, 18 were verified among 15 participants, with 9 (50%) verified among patients with type A/B footwears, 8 (44.4%) with type C footwears, and 1 (5.6%) in patients with type D footwears (Table 3). Classified according to Gustillo and Anderson, there was a general predominance of grade 2 injuries (46.7%), and a greater association with type A/B footwears, but not statistically significant (p = 0.895) (See Table 4).

From the sample, 28 patients required hospitalization (38.9%), with no significant relationship verified with the type of footwear (p = 0.393) (Table 4), the highest number of hospitalizations was related to patients with type A/B footwears (38.3%), with the relative value lower for type D patients (22.2%). The length of hospitalization also showed no significant difference between the evaluated groups (p = 0.577) (Table 5).

**Table 5:** Length of hospitalization and number of surgical procedures by participants’ footwear type.

Variables	Footwear A/B	Footwear C	Footwear D	P value
**Length of hospitalization**				**0.577**
Mean ± standard deviation	2.9 ± 5.2	3.6 ± 5.0	3.2 ± 6.4	
Median (1st - 3rd quartile)	0.0 (0.0 - 3.0)	0.5 (0.0 - 5.5)	0.0 (0.0 - 0.0)	
**Number of surgical procedures**				**0.355**
Mean ± standard deviation	0.6 ± 0.7	0.8 ± 1.0	0.3 ± 0.7	
Median (1st - 3rd quartile)	0.0 (0.0 - 1.0)	0.5 (0 - 1.5)	0.0 (0.0 - 0.0)	

Kruskal-Wallis test.

Additionally, 44.4% (32 patients) of participants required some surgical procedure, totaling 42 procedures, distributed by footwear type (Table 3). There was a higher number of procedures in participants with type A/B footwears (61.9%).

Regarding the need for surgical procedures, a lower trend was observed among participants with type D footwears (77.8% did not require surgical intervention) (p = 0.432) (Table 4). No significant relationship was observed between the number of surgeries and the type of footwear used (p = 0.355) (Table 5).

## DISCUSSION

After consolidating and analyzing the data, a dominance of male patients (77.8%) was observed, consistent with epidemiological data in the DPVAT report (2018)^15^. The predominant age group was between 20-29 years (30.6%), with an average age in the sample of 34.2 years (+/- 14.5), showing a peak incidence of trauma in young patients, consistent with Brazilian references.^15^

Regarding the use of footwears, there was a predominance of type B (62.5%), with only 2 participants not wearing any footwear (2.78%). It is noteworthy that there was no significant relationship in the analysis of patients with type A footwear due to the limitations of the sample. Despite Article 232 of the Brazilian Traffic Code,^16^ which prohibits and fines motorcycle riders who wear footwears that does not secure their feet, category B, which precisely includes these types of footwears, was the most prevalent, highlighting the lack of or low enforcement by competent authorities, as well as the lack of engagement of the population with the rule. However, the same legislation does not determine which protection option is more effective, leaving it to the rider to choose. For cultural and economic reasons, especially in the northeastern region, there is a strong inclination towards the everyday use of espadrilles or sandal-type footwear, which explains the preference for this specific type. Taking this into account, the analyses were conducted by combining the samples from groups A and B, generating group A/B, since the types of protection offered by these two groups are similar.

Approximately 2/3 of the participants presented soft tissue injuries without exposure (68%), with or without fractures, and the highest number of injuries was in group A/B (41.7%), and the lowest was in group D (11.1%). The injuries were predominantly low-grade – 77.5% were AOSTC-IC < 3. A higher frequency and severity of closed soft-tissue injuries were observed among participants using type A/B footwear, with >30% of injuries graded ≥3 (p = 0.004). Participants wearing footwears with greater protection, in turn, correlated with a lower number of injuries, and these with a lower grade, with neither of these two groups presenting any injuries classified by AOSTC-IC 3 or higher.

These results were corroborated by categorizing the injuries according to the Tscherne classification, in which a significant difference was observed (p < 0.001) in the same direction. Participants with type A/B footwears presented a higher number of soft tissue injuries in closed trauma (61.2%), and with greater severity – 26.7% of the injuries in the group were grade 2. When compared with groups C and D, these presented all their injuries classified as ≤ 1.

Among patients with sprain-type injuries, the majority were grade 2 (64.0%). Although there was a relatively higher frequency of these injuries (76% of the total) and greater severity (68.4% grade 2) with group A/B, it was not possible to verify statistical significance between the occurrence of sprains (p = 0.458) or the grade of the injury (p = 0.803) according to the type of footwear.

Similarly, exposed injuries with or without fractures showed a relatively higher prevalence in participants from group A/B (36.2%), and the lowest prevalence was in group D (11.1%). However, such values did not show a significant difference (p = 0.388). It was also not possible to verify a significant difference in these injuries with the type of footwear, when classified by AOSTC – IO (p = 0.308).

The trends observed in both sprains and exposed injuries, although not statistically significant, suggest a possible protective factor that varies by footwear. This explanation is likely due to the low number of individuals participating in the study. This also seems to apply to the other data to be discussed below.

The prevalence of closed and exposed fractures and/or dislocations was higher in patients from groups A/B (42.8% and 50%, respectively) and lower in participants from group D (5.5% and 5.6%, respectively). There was no significant difference in the severity of exposed fractures by type of footwear. Again, with a low statistical correlation of these data, hindered by the sample size.

The data also showed no significant differences in the number and duration of hospitalizations across the groups, as well as in the need for and number of surgical procedures.

The predominance of forefoot fractures over others is likely caused by being the most exposed area of the foot, being more distal and closer to the ground during motorcycle use, and also, because the predominant type of footwear is type B, which does not provide a significant means of staying secured to the foot during an accident/trauma, resembling, in trauma, an individual barefoot, similar to group A.

What can be postulated, considering the predominance of patients using type B footwears with a progressive numerical reduction of types C and D, is that there is a possibility that these individuals (groups C and D) were not even classified with an injury in the lower limb, due to the use of footwear with greater protective power, or that they did not even seek care in the emergency room in cases of lower energy trauma, thus leading to a sampling bias. Another consideration is that groups C and D only seek care in cases of higher energy trauma, making it difficult to identify the protective factor of the footwear. Furthermore, it may be due to low usage for cultural reasons. Such a problem can be rectified and consequently corrected by changing the way participants are selected, including all motorcycle accident victims, and not just those presenting with any injury in the lower limb, and/or differentiating levels of trauma energy.

Another possible bias is that, in high-energy traumas, the use of traditional footwears, regardless of the level of protection, does not provide sufficient protection to alter the degree of injury.

Still, the analysis of the data suggests a progressive relationship between the number of injury severity according to the level of protection at the time of trauma. Individuals who wore footwears without adequate coverage of soft parts and without closed protection at the level of the dorsum of the foot and ankle were relatively more affected. Although no correlating increase was verified in the frequency and duration of hospitalization, as well as in the number of surgical procedures required.

## CONCLUSION

The results of the present study showed that the majority of the population uses footwear considered inadequate for riding motorcycles according to current traffic legislation, and that there is a relationship between this type of footwear and a higher prevalence of soft tissue injuries, while patients using footwear with protection above the ankle were the most protected. There was still a trend toward greater protection against sprains, fractures, and open injuries with increasing footwear protection, but it was not statistically significant. Despite the above, there was no statistical significance regarding the need for and duration of hospitalization and surgeries.

## Data Availability

The contents underlying the research are available in the manuscript.
